# From commensalism to pathogenesis: the hidden role of the respiratory virome

**DOI:** 10.3389/fcimb.2025.1693796

**Published:** 2025-10-16

**Authors:** Zhihao Wang, Lei Song, Dan Li, Yuanhui Jin

**Affiliations:** ^1^ Department of Geriatrics, Jilin Geriatrics Clinical Research Center, The First Hospital of Jilin University, Changchun, China; ^2^ Department of Respiratory Medicine, Center for Pathogen Biology and Infectious Diseases, Jilin Provincial Key Laboratory for Individualized Diagnosis and Treatment of Pulmonary Diseases, The First Hospital of Jilin University, Changchun, China; ^3^ Meihekou Central Hospital, Meihekou, China

**Keywords:** asthma, pulmonary fibrosis, microbiota, phage, pneumonia

## Abstract

The respiratory virome, encompassing both eukaryotic viruses and bacteriophages, is an essential but often overlooked component of the airway microbiome. Recent advances in metagenomics have revealed that a diverse viral community exists even in healthy individuals, contributing to immune regulation and microbial balance. However, the field faces several challenges: the baseline composition of the respiratory virome remains incompletely defined, its immunomodulatory functions are not fully understood, and its contributions to respiratory diseases are only beginning to be elucidated. This mini-review summarizes current knowledge of the respiratory virome under physiological conditions, highlights emerging insights into how resident viruses and phages shape host immunity, and discusses alterations observed in asthma, chronic obstructive pulmonary disease, pulmonary fibrosis, and pneumonia. By integrating evidence across these conditions, we emphasize the significance of the virome in both health and disease. A deeper understanding of its dynamics may yield novel diagnostic markers and therapeutic strategies, underscoring the importance of future longitudinal and mechanistic studies in this rapidly evolving field.

## Introduction

The virome is defined as the viral component of the human microbiome, comprising the genomes of all viruses in a body site – including eukaryotic viruses (infecting human cells), bacteriophages (infecting bacteria), and endogenous viral elements (Porto, 2022). Historically, viral communities in the lungs were understudied due to technological limitations, but advances in deep sequencing and metagenomics have revealed that the healthy respiratory tract harbors a complex virome (Porto, 2022; Conradie et al., 2024). A recent meta-analysis identified ~320 distinct viral species across 26 families in the human virome, with *Anelloviridae* (anelloviruses), *Papillomaviridae*, and *Bunyaviridae* among the most abundant families overall (Rodrigues et al., 2017). Many of these are “commensal” or low-level viruses that establish persistent or transient infection without causing acute disease (Koonin et al., 2021).

In the lung, this basal virome includes DNA and RNA viruses as well as bacteriophages, which colonize the nasopharynx, bronchi and alveoli (Sandybayev et al., 2022; Li et al., 2024). For example, metagenomic surveys detect common respiratory viruses (rhinoviruses, respiratory syncytial virus, adenoviruses, herpesviruses), anelloviruses (e.g. torque teno viruses), papillomaviruses, and a wide array of phages targeting resident bacteria[1][16]. In one large analysis of public lung sequencing data, researchers assembled 1842 viral contigs (complete/near-complete genomes) across 25 families (Conradie et al., 2024). Notably, bacteriophages often dominate the recovered sequences– reflecting the high diversity of the gut-like phageome in the airways (Dodi et al., 2021; Conradie et al., 2024). For instance, active bacteriophages corresponding to bacterial respiratory pathogens can be detected—and in some cases isolated—from human lower-airway specimens (e.g., sputum, BALF), most robustly exemplified by Pf filamentous phages of *Pseudomonas aeruginosa* in cystic fibrosis (Brown-Jaque et al., 2018; Agudelo-Romero et al., 2025; Burgener et al., 2025), and even typical gut phages (crAssphages) have been detected in respiratory samples (Camarillo-Guerrero et al., 2021; Conradie et al., 2024). Overall, these findings suggest that phages are a major component of the respiratory microbiome and likely enter the lung via micro-aspiration or gastroesophageal reflux.

**Figure 1:**
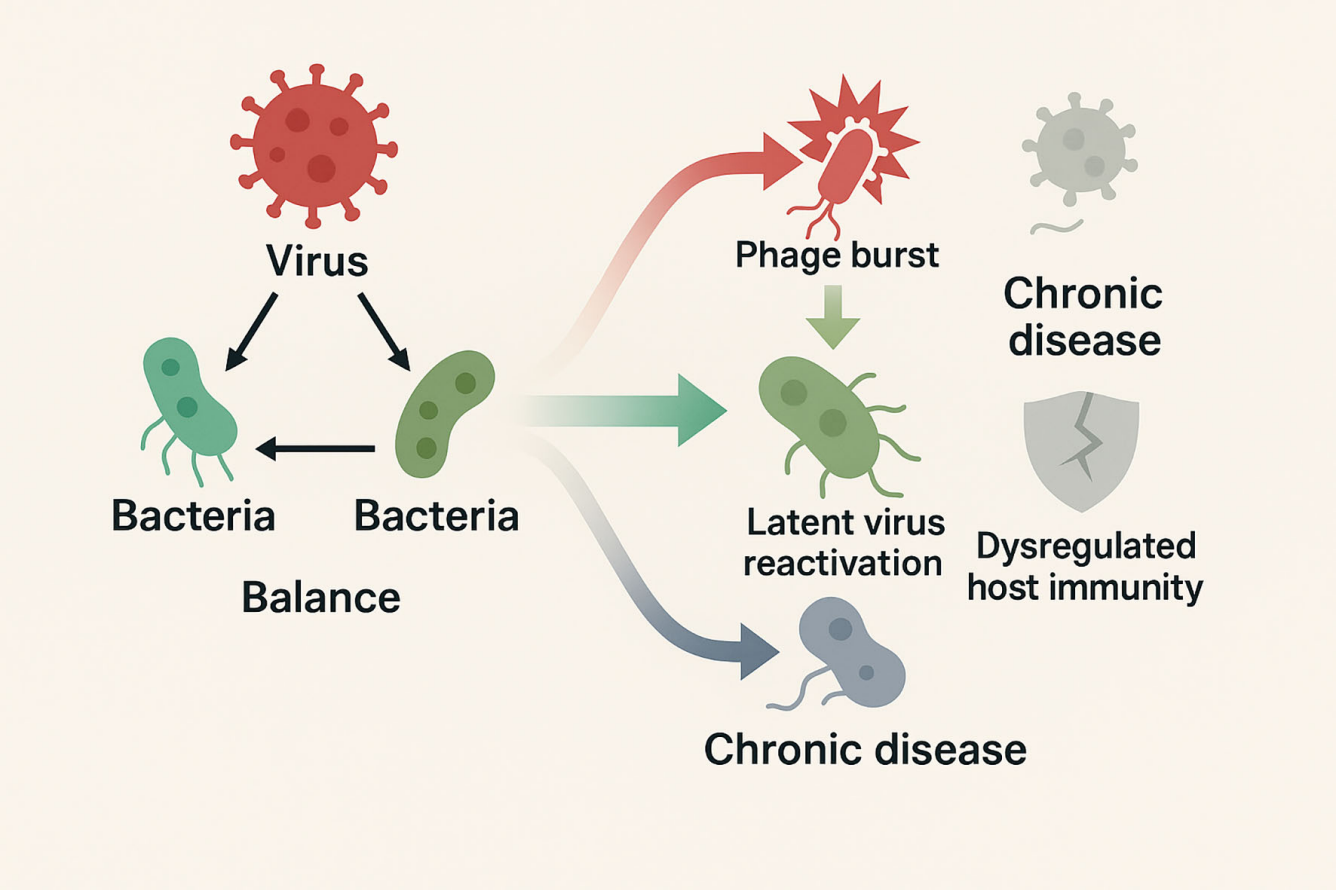
Schematic illustrates the balanced virus–phage–host immunity network in health, contrasted with acute (viral bursts, phage restructuring, latent virus reactivation to inflammation storm) and chronic (low-level persistent infection, phage decline, immune dysregulation to fibrosis and remodeling) disease states.

Importantly, the healthy lung virome is not inert. Its low-level viral inhabitants continuously engage the immune system and can confer protection. Subclinical colonization by diverse viruses—collectively termed the ‘residential virome’—may contribute to the tonic priming of innate antiviral immunity in the airways (Porto, 2022). By contrast, when viral burden rises or the immune response falters, this balance is disturbed, potentially triggering or worsening inflammatory disease (Porto, 2022). In this review, we outline the typical composition of the respiratory virome in health, examine how it modulates host immunity, and then adopt an acute versus chronic respiratory disease framework, providing a thematic lens to highlight both contrasts and overlaps in virome dynamics across respiratory disorders (**Figure 1**).

## The normal respiratory virome

The normal respiratory virome in healthy humans is a complex ecosystem primarily dominated by bacteriophages along with a smaller, yet biologically significant, component of eukaryotic viruses. Bacteriophages, which are central to this ecosystem, regulates bacterial population dynamics through lytic and lysogenic cycles and affecting bacterial gene transfer, including the spread of virulence factors and antibiotic resistance genes (Jankauskaite et al., 2018; Conradie et al., 2024). Phage families commonly detected include Siphoviridae, Myoviridae, and Podoviridae, members of the order Caudovirales, as well as Microviridae (Gregory et al., 2018; Jankauskaite et al., 2018). These phages are thought to contribute to the maintenance of a balanced bacterial community within the respiratory tract and may indirectly support the host’s defense against opportunistic pathogens (Mitchell et al., 2016).

Eukaryotic viruses in the respiratory virome include several taxa that persist in a nonpathogenic state in healthy individuals. Among these, anelloviruses—particularly Torque Teno Virus (TTV) and related species—are consistently reported as abundant components and are considered potential biomarkers of immune status (Dodi et al., 2021; Porto, 2022). Other eukaryotic viruses detected in healthy respiratory secretions comprise members of the herpesvirus, adenovirus, papillomavirus, and paramyxovirus families, as well as respiratory syncytial virus (RSV) and rhinoviruses that are present at low levels possibly due to asymptomatic colonization (Mitchell et al., 2016; Wylie, 2017). In addition, metagenomic studies have occasionally identified viruses of gastrointestinal origin in airway samples, suggesting that micro-aspiration or translocation events may occur (Conradie et al., 2024; Li et al., 2024).

In summary, a healthy respiratory tract hosts a complex virome: a mélange of occasional pathogens, latent viruses, and especially a rich phage community. These resident viruses help define a “baseline” airway environment. The next section discusses how this baseline virome interacts with and regulates the immune system.

## Acute respiratory diseases

Acute respiratory diseases are characterized by sudden infection and inflammation, often with rapid pathogen overgrowth. Here we focus on pneumonia as a representative acute disease and describe how the airway virome shifts during infection.

## Pneumonia

Recent studies have shown that pneumonia is accompanied by significant shifts in the respiratory virome, including eukaryotic viruses, bacteriophages, and endogenous retroviruses. Both pediatric and adult cohorts demonstrate that pneumonia onset correlates with altered virome diversity and composition compared to healthy states. For example, a metagenomic survey of young children in South Africa revealed that those with severe acute respiratory infections (SARI) harbored a higher abundance and diversity of viruses than controls, including ubiquitous rhinoviruses and sequences of human endogenous retrovirus K113, while pathogenic Pneumoviridae (e.g., RSV) were detected exclusively in pneumonia cases (Ogunbayo et al., 2022). These findings imply that perturbations in the viral ecosystem—loss of benign virome members and surges of others—may contribute to pneumonia development rather than merely reflecting it. Similarly, loss of microbial complexity under ICU stress, such as during mechanical ventilation, precedes overgrowth of pathogens; while bacterial changes are well documented, the viral and fungal components are less explored (Fromentin et al., 2021). Collectively, these results highlight that virome disturbances are early harbingers of pneumonia across age groups.

Once pneumonia is established, dynamic virome shifts appear to influence disease severity. In children, metagenomic characterization has revealed distinct viral assemblages associated with pneumonia. Cui et al. identified over 1,100 viral taxa differing between pneumonia cases and healthy controls: phages infecting commensals such as Prevotella, Neisseria, and Veillonella were reduced, while adenoviruses, polyomaviruses, and phages targeting Streptococcus and Staphylococcus were enriched (Cui et al., 2024). Notably, some adenovirus and polyomavirus infections at high abundance were missed by PCR-based testing, underscoring the diagnostic value of virome profiling. Co-infections further shape outcomes: in a Beijing cohort, over half of children with *Mycoplasma pneumoniae* pneumonia were co-infected with respiratory viruses, most often influenza or RSV, leading to prolonged fever and hospitalization (Guo et al., 2022). A more recent analysis showed school-aged children with *M. pneumoniae* pneumonia had an expanded virome, with enrichment of DNA virus families (Poxviridae, Retroviridae, Iridoviridae) and shifts in phage families (rise of Myoviridae, decline of Siphoviridae), along with detection of rhinovirus and respirovirus in lower airways absent in controls (Zhang et al., 2025). Similar trends were observed in Mexico, where pneumonia patients’ viromes were dominated by torque teno mini-virus and a Streptococcus phage (EJ-1), while asthma patients were dominated by RNA viruses like rhinovirus and RSV (Romero-Espinoza et al., 2018). Together, these pediatric studies suggest that bacterial or viral pneumonia disrupts the resident virome, enabling expansion of latent or uncommon viruses and altering phage–host interactions.

In adults, pneumonia progression often involves latent virus reactivation and a collapse of viral diversity. More recently, metagenomic sequencing confirmed herpesvirus predominance in critically ill COVID-19 ARDS patients, where CMV and HSV-1 were found in nearly all cases (Mitsuyama et al., 2025). Strikingly, endogenous retroviral elements are also activated in severe pneumonia: HERV-K transcripts were significantly elevated in tracheal aspirates of critically ill COVID-19 patients compared to mild cases or non-COVID controls, correlating with IL-17–rich inflammation, monocyte activation, and early ICU mortality (Temerozo et al., 2022). *In vitro*, SARS-CoV-2 infection of monocytes directly induced HERV-K expression, implicating endogenous retroviruses as amplifiers of immunopathology. Pediatric samples also show HERV-K113 in both SARI and healthy children (Ogunbayo et al., 2022), though a pathogenic role is less clear. Beyond human viruses, severe pneumonia has been linked to enrichment of exogenous viruses: metatranscriptomic analysis of COVID-19 lungs revealed unexpected detection of plant Tobacco mosaic virus exclusively in patients, alongside Klebsiella overgrowth and loss of benign taxa, reflecting extreme dysbiosis (Han et al., 2022). Thus, in severe pneumonia, latent and endogenous viruses “awaken”, compounding lung damage and inflammation.

Bacteriophages are increasingly recognized as mediators of pneumonia-associated microbial imbalance. In children with *M. pneumoniae* pneumonia, phage shifts paralleled bacterial changes: expansion of lytic phages (Myoviridae, Ackermannviridae) and decline of temperate phages (Siphoviridae) mirrored bacterial overgrowth (Zhang et al., 2025). In Mexico, pneumonia patients showed enrichment of anelloviruses and Streptococcus phage EJ-1, whereas asthma cases were dominated by RNA respiratory viruses (Romero-Espinoza et al., 2018). These findings support a model where phages destabilize bacterial communities, clear niches for opportunistic pathogens, or transfer virulence genes, thereby facilitating pneumonia. Overall, pneumonia reflects a breakdown of virome–microbiome–host equilibrium: eukaryotic viruses dominate, phages restructure bacterial ecosystems, and endogenous retroviruses activate, all amplifying inflammation and tissue damage. Understanding these dynamics offers potential for novel biomarkers and therapeutic strategies aimed at restoring virome balance.

## Chronic respiratory diseases

Chronic respiratory diseases are characterized by persistent airway inflammation and progressive remodeling. In these conditions, viral infections can act as triggers or modulators of pathogenesis. Below, we discuss how the airway virome is altered in chronic diseases such as asthma, COPD, and pulmonary fibrosis.

## Asthma

Respiratory viruses are recognized as major triggers for asthma exacerbations. HRV, in particular, is the most commonly detected virus during asthma attacks and has been implicated in the development of persistent wheezing, especially when infection occurs during early life (Freer et al., 2018; Lejeune et al., 2020). Rhinovirus infections promote airway inflammation through the induction of mucin gene expression and the release of pro-inflammatory cytokines that compromise epithelial barrier function (Jankauskaite et al., 2018; Romero-Tapia et al., 2023). In asthmatic individuals, these viral infections are associated with a skewing of the immune response toward a Th2 phenotype, with increased production of IL-4, IL-5, and IL-13 that contribute to eosinophilic inflammation (Lejeune et al., 2020; Romero-Tapia et al., 2023). RSV is another prominent virus in early childhood whose infections have been linked to subsequent asthma risk, possibly through its capacity to alter neonatal lung development and immune maturation (Lejeune et al., 2020; Liu et al., 2025). Furthermore, influenza virus and parainfluenza provide additional triggers that not only exacerbate asthma but also may interact with emerging viral families such as Anelloviridae to modulate disease severity (Jankauskaite et al., 2018; Romero-Tapia et al., 2023). Evidence also suggests that specific viral subtypes, such as rhinovirus C, exhibit enhanced pathogenicity in predisposed individuals via receptor-mediated cellular entry and subsequent robust inflammatory responses (Jankauskaite et al., 2018; Lejeune et al., 2020).

Recent studies have provided compelling evidence for a distinct pattern of virome dysbiosis in asthmatic airways. In asthmatic children, metagenomic surveys have identified a significant reduction in the abundance and diversity of bacteriophages, which correlates with an expansion of eukaryotic viruses, particularly those of the Anelloviridae and Picornaviridae families (Megremis et al., 2023). This altered viral ecology is hypothesized to compromise the regulatory functions of bacteriophages, leading to disordered bacterial communities and a less resilient airway microbial ecosystem (Megremis et al., 2023; Xepapadaki et al., 2024). Detailed investigations have stratified the virome into distinct profile groups; for instance, the “Prokaryotic Virome Profile Group” (PVPG) is more prevalent among healthy children, whereas groups characterized by high eukaryotic viral richness or dominated by Anelloviridae (e.g., the AVPG) are overrepresented in asthmatic cohorts and tend to correlate with poorer asthma control and increased severity (Megremis et al., 2023). These findings underscore that the restructuring of the airway virome may be both a consequence of, and a contributing factor to, the chronic inflammatory milieu observed in asthma (Megremis et al., 2023; Walsh et al., 2023).

## COPD

COPD is characterized by irreversible airflow limitation and chronic inflammation. In patients with COPD, a diverse array of respiratory viruses has been detected, ranging from common RNA viruses—such as rhinovirus, influenza virus, respiratory syncytial virus (RSV), parainfluenza, and coronavirus—to DNA viruses including adenoviruses, Epstein–Barr virus (EBV), and cytomegalovirus (CMV) (Britto et al., 2017). Notably, latent viral infections have also been documented; for example, adenoviral E1A protein expression and persistent EBV presence have been linked with increased inflammatory responses and faster declines in lung function (Guo-Parke et al., 2020; D’Anna et al., 2021). Moreover, emerging molecular studies have identified the prevalence of anelloviruses, particularly torque teno virus (TTV), as a significant component of the airway virome in COPD patients, with relative abundances that differ markedly from those observed in healthy subjects (van Rijn et al., 2019). In addition to pathogenic viruses, the airway ecosystem also includes bacteriophages such as members of the Siphoviridae, Myoviridae, and Podoviridae families that may influence the bacterial microbiome through processes such as horizontal gene transfer and modulation of bacterial virulence (Linden et al., 2019; D’Anna et al., 2021). This intricate blend of viral species forms a dynamic community whose composition is subject to the influence of host factors, environmental exposures, and therapeutic interventions (Linden et al., 2019).

Overall, the COPD virome seems perturbed in ways that mirror other chronic lung diseases: pathogenic viruses surge during exacerbations, and low-level viruses (anelloviruses, herpesviruses) accumulate with disease progression, while protective phage communities wane. These shifts amplify innate inflammation in the airway epithelium and drive disease exacerbations.

## Pulmonary fibrosis

Respiratory viruses have long been considered potential triggers in the development of idiopathic pulmonary fibrosis (IPF) in genetically susceptible individuals. In particular, Epstein–Barr virus (EBV) and other herpesviruses have been frequently detected in IPF patients (Ntolios et al., 2021). However, none of these studies could establish a direct causal relationship between EBV and fibrosis (Ntolios et al., 2021). Some reports linked hepatitis C virus (HCV) infection to IPF, raising the possibility that HCV could contribute to fibrotic lung disease (Ntolios et al., 2021). Nevertheless, findings have been inconsistent, as other analyses found no higher HCV prevalence among IPF patients (Irving et al., 1993). Moreover, modern broad-range virome analyses have not identified a unique viral signature in IPF. A next-generation RNA sequencing survey of lung tissue (screening for hundreds of viruses) detected only sporadic viral sequences and showed no significant difference in total viral burden between IPF lungs and control lungs (Yin et al., 2020). Consistently, a recent meta-analysis reported that while more than half of IPF patients have evidence of viral infection – especially herpesviruses like EBV and HSV – these viruses are also prevalent in the general population, and a definitive etiologic role in fibrosis remains unproven (Valenzi et al., 2021).

In the context of acute exacerbations of IPF (AE-IPF), the lung virome appears to play a more pronounced role. Wootton et al. used molecular assays to detect viruses in the lungs and found viral genetic material in approximately 44% of patients experiencing an acute IPF exacerbation, whereas none of the stable IPF patients harbored detectable virus at that time (Wootton et al., 2011). Among those exacerbation cases, torque teno virus (TTV) was frequently identified (present in about one-third of AE-IPF patients) and its presence has been associated with worse survival outcomes in IPF (Wootton et al., 2011). Similarly, another study observed that 57 different viral species in nasopharyngeal samples from AE-IPF patients, with herpesviruses and influenza A being the most common, and overall viral detection was significantly more frequent in acute exacerbations than in stable disease (Weng et al., 2019). These observations support the idea that viral infections can precipitate acute lung injury on a background of chronic fibrosis, thereby accelerating the clinical decline. Still, it is important to note that no single virus is consistently found in all exacerbations, and many exacerbations have no identifiable pathogen – indicating that viruses are one of several possible triggers.

In summary, while IPF lungs show ecological disturbance (microbial dysbiosis) and susceptibility to infection, no single virus has been singled out as causal. The virome in IPF likely reflects an environment of impaired immunity and injured epithelium: as fibrosis progresses, alveolar barriers break down, allowing greater microbial ingress (including viruses). Higher viral loads (as part of increased total burden) may worsen inflammation in advanced IPF, but evidence for direct virus-driven fibrosis is lacking. Thus, unlike asthma or COPD, pulmonary fibrosis appears not to require a specific viral trigger in most cases.

## Conclusion and future perspective

The respiratory virome is an integral yet underappreciated element of pulmonary ecology. In health, a rich and balanced virome – comprised of both eukaryotic viruses and a diverse phageome – helps maintain immune homeostasis and microbiome equilibrium. The examples above show that alterations in virome composition often accompany respiratory diseases: asthma is characterized by viral overgrowth and phage loss; COPD by recurrent viral provocations on a background of virome shift; pneumonia by dominance of pathogens and reduced viral diversity; and pulmonary fibrosis by overall microbial burden without a specific viral signature. These observations underscore that the virome should be considered alongside bacteria and fungi in pathogenesis models.

However, many questions remain. Causality is difficult to establish: does a disturbed virome drive disease, or does disease (and its treatments) reshape the virome? Longitudinal cohort studies are needed to disentangle cause and effect. Further, most virome analyses to date rely on DNA sequencing and are biased against RNA viruses – yet many respiratory pathogens are RNA viruses. Improved metatranscriptomic approaches will better capture the full virome. Functional studies are also needed. Can we experimentally manipulate the virome (e.g. phage therapy or controlled virus exposures) to alter disease outcomes? For instance, inhaling benign viruses that boost interferon might protect high-risk patients from severe infections. Conversely, targeting deleterious viruses (like persistent anellovirus or herpesvirus populations) might reduce chronic inflammation in asthma. In practical terms, virome profiling may yield biomarkers for disease. The cited studies suggest that specific virome “fingerprints” correlate with asthma severity and pneumonia etiologies. Future diagnostics might include virome sequencing of sputum or nasal swabs to stratify patients. More speculatively, engineered phages could one day be used to reshape lung bacterial communities in chronic disease.

In conclusion, the respiratory virome is a dynamic ecosystem whose balance is linked to lung health. Recent research has begun to unveil its complexity and roles, but the field is still young. As methods improve, integrating virome data with microbiome and host immune data will be crucial. Deeper understanding of how viruses and phages influence the respiratory tract promises not only new scientific insights but also novel therapeutic strategies for asthma, COPD, fibrosis, pneumonia, and beyond.
